# The effect of plastic tape seal to reduce face seal leak in respirator N-95 type 1860

**DOI:** 10.1016/j.amsu.2022.104287

**Published:** 2022-08-10

**Authors:** Mirta Hediyati Reksodiputro, Harim Priyono, Luthfi Ari Wibowo, Jenny Bashiruddin, Ratna Dwi Restuti, Dini Widiarni Widodo, Dewi Sumaryani Soemarko, Joedo Prihartanto, Prasandhya Astagiri Yusuf

**Affiliations:** a Department of Otorhinolaryngology-Head and Neck Surgery, Faculty of Medicine Universitas Indonesia/Dr. Cipto Mangunkusumo Hospital, Jakarta, Indonesia; b Department of Community Medicine, Faculty of Medicine Universitas Indonesia/Dr. Cipto Mangunkusumo Hospital, Jakarta, Indonesia; c Department of Medical Physic, Faculty of Medicine Universitas Indonesia/Dr. Cipto Mangunkusumo Hospital, Jakarta, Indonesia

**Keywords:** Respirator leak, Plastic tape, Bitrex fit test, N-95 respirator type 1860

## Abstract

The risk of face seal leak while using N-95 respirators is experienced by health workers and thus failing fit test are quite common. Finding solutions to overcome face seal leaks is crucial; one of which is by sealing the N-95 respirator. The seal used in this research was Tegaderm® a transparent film dressing or a plastic tape which is known to have the advantages of strong adhesion, high level of pore density and standardized medical grade. This study tries to determine the effectiveness of plastic tape adhesive on the N-95 type 1860 respirator in overcoming face seal leak qualitative fit test using Bitrex immediately after being worn and after 4 h of using it. The study used a quantitative approach with an incidence study design conducted pre and post experimental without comparison to see the effectiveness of plastic tape sealing. The subjects for the research were 81 health workers in the CMH environment who were at risk of being exposed to COVID-19. The study found a significant difference in the Bitrex fit test immediately after sealing the N-95 type 1860 respirator with plastic tape; 100% passed the fit test immediately after sealing, and 64.2% passed the fit test after 4 h of working. The effectiveness of sealing using plastic tape is considered to be quite good to overcome face seal leak on the N-95 type 1860 respirator. Health care workers need to be more vigilance to ensure better face seal.

## Highlights

• There is an increase need of protection against aerosol born disease (COVID-19).
• Disruption in face seal when using respirator increases exposure.
• Utilisation of plastic tape eliminate face seal leak while using N-95 respirator.

## 1 Introduction

The COVID-19 pandemic has resulted in scarcity of personal protective equipment for health care workers (HCWs) eventhough it is critical for their safety. Some medical procedure especially in aerosol generating procedure, require its users the respiratory protection against the aerosol borne diseases like COVID-19. World Health Organisation (WHO) and Centers for Disease Control and Prevention (CDC) recommend the use of N95 respirators certified by National Institute for Occupational Safety and Health (NIOSH) for the health care workers dealing with patient suspected with aerosol borne diseases [1]. However, according to Pilot Study by Wardhan [2] et al. approximately 30% of HCWs fail the qualitative fit test (QLFT) for N-95, thus making them at risk of infection transmission. The lack of seal increases exposure to aerosolized droplets as for respirators, it enters through breaks in the face seal, suggesting that better mask to face seal should be achieved to improve the efficacy of the filter medium [3].

The face seal leak problem has been the subject of research over the past decade. Recently qualitative fit test (QLTF) of the N-95 respirators at the University of Florida which was performed during the COVID-19 pandemic showed that 68% of HCWs who previously failed QLTF with their first choice respirators passed the retest with 3 M N-95 respirators type 1860 and 1860S modified with adhesive along the perimeter of the respirator [2].

In a previous pilot study by the author, HCWs who failed the qualitative saccharin fit test was retested with modification of medical grade adhesive (hypafix®) to seal the entire edge of the respirator. It was found that 60% of HCWs who used the respirator with adhesive modification passed the retest and none of the medical grade adhesive had been tested for sealing respirators. In this study we used Tegaderm® (plastic tape) is because it is widely available in various health services, is easy to use, has a stronger bond, has more density and is more water resistant when compared to other types of adhesives. We hypothesized that plastic tape as an adhesive seal along the edges of a respirator will improve its effectiveness.

This study aims to inform all health care workers about the importance to use N-95 respirators properly and correctly, as well as the awareness of the risk of face seal leak in the use of N-95 respirators which can endanger health workers.

## 2 Methods

This study try to look upon a solutions for health care worker who previously failed respirator fit tests with 3 M N-95 respirators type 1860 by sealing N-95 respirators with medical grade transparent film dressing (Tegaderm). A pilot study with a modification of 3 M N-95 respirators type 1860 sealed with other medical grade adhesive (Hypafix) was done earlier to test the efficacy of medical tape in achieving a better face seal. This experimental study aims to analyze the effect of plastic tape to seal respirator to face immediately after the Bitrex fit test and perform a follow up analysis 4 h post-activity. The study was conducted at the ENT-Head and Neck specialist clinic, Inpatient Care Building and Kiara (COVID Care Unit) Cipto Mangunkusumo Hospital. The study started after passing the ethical review (KET-171/UN2.F1/ETI/PPM.February 00, 2021) from the committee for Medical Research Ethics/Health FMUI/Cipto Mangunkusumo. The study was carried out for 6 months (February–July 2021). In this study, the sample size was observed using non-probability sampling with consecutive sampling method.

**Fig. 1:**
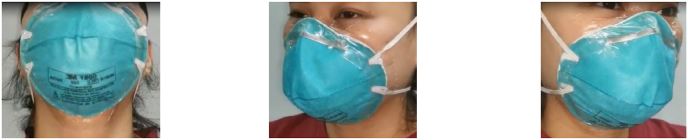
Medical grade plastic tape attached around the perimeter of N-95 tipe 1860.

The target population in this study were the medical officers at Cipto Mangunkusumo Hospital who used N-95 type 1860 respirators. Each participant who failed the fit test with Bitrex was retested using modification of respirator with adhesive plastic tape, a transparent medical dressing made of a thin polyurethane membrane coated with an acrylic adhesive layer (Tegaderm; 3 M) which was adhered around the N-95 type 1860 respirator. The adhesive sealing tape was cut in 3 × 6 cm size and sealed around gap between face and respirator. The sealing was done by overlapping the ends of the plastic tape around the perimeter of respirator (Fig. 1).

**Fig. 2:**
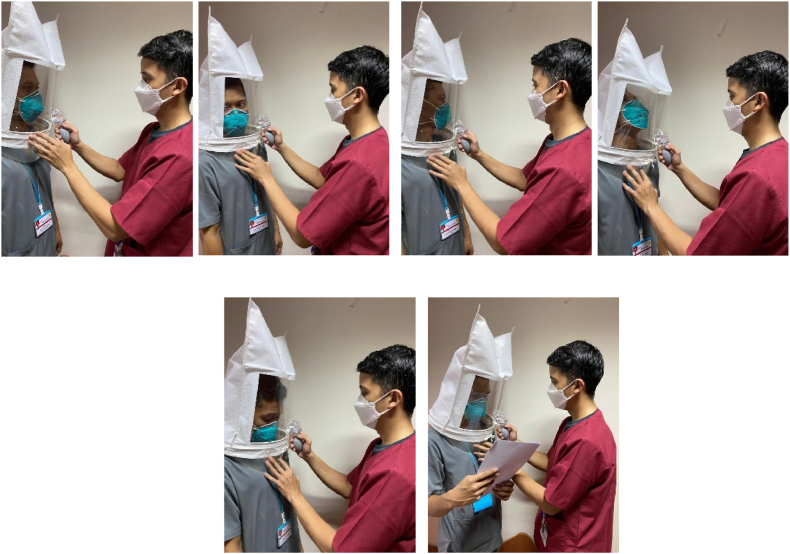
Fit test Procedure.

For the Qualitative Fit Test (QLTF), we used the Bitrex 3 M Qualitative Fit test Apparatus FT-3) solution aerosol protocol as described by the Occupational Safety and Health Administration of United States of America. When using the respirators, the participants were exposed to the injection of aerosol solution of Bitrex under the hood and were instructed to perform several activities such as: breathing normally, deep breathing, turning head side to side, moving head up and down, and reading one paragraph of a reading text (Fig. 2). Participants were considered to pass the fit test if they did not detect a bitter taste at any time during the test. The respirator used by the participants who passed the fit test was considered adequate to give respiratory protection to its users. The Bitrex fit test was repeated after the subjects had used the sealed respirator under normal activities during their work in ward or clinic for 4 h.

### 2.1 Statistical analysis

Statistical data management was performed by SPSS (Statistical Package for Social Sciences) version 20.0 software. Continous data from the demographic data were summarized using mean and standard deviation (SD), whereas the categorical data were listed using percentages. Chi-square test was performed to see the difference in proportions and the relationship between the independent variable and the dependent variable as well as to identify significant variables. Linear regression test was used to determine the association of the independent variables and to identify the most influential dependent variable.

## 3 Results

**Fig. 3:**
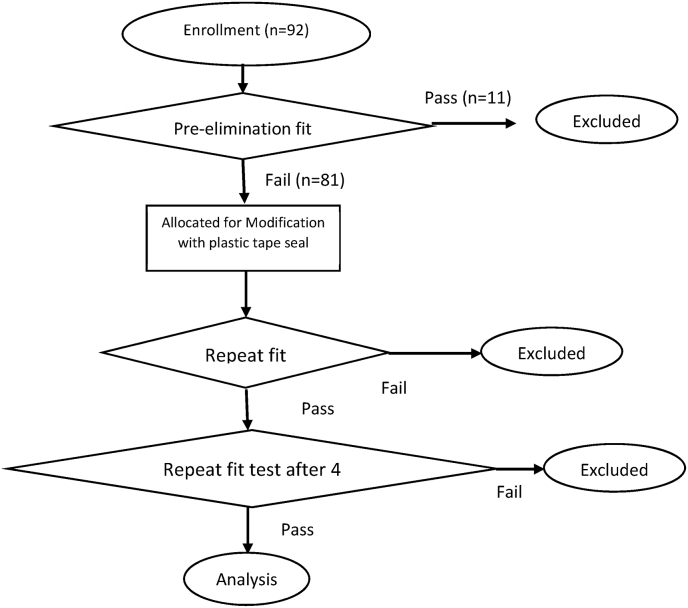
Flow diagram of sample recruitment.

From a total of 92 subjects, 11 participants were excluded from the study because the participants had passed the fit test; thus, they were not eligible to go through the next part of the study which assess the efficacy of modified adhesive respirator to seal the mask for health care workers whose previously failed the fit test. Eighty one participants were retested using N-95 respirator type 1860 sealed with plastic tape (Fig. 3). The following list is the characteristics of the research subjects (Table 1).

**Table 1:** Characteristics of subjects based on gender, age group, age, type of work, workplace, and symptoms of Ageusia.

Characteristics of Subjects	Fail Fit Test	Percentage
Gender		
Male	13	16
Female	68	84
Age group		
21–40 years	68	83,9
41–55 years	13	16,1
Type of work		
Nurse	69	85,2
Intern	1	1,2
Doctor	11	13,6
Workplace		
ENT Specialist Clinic	9	11,1
Inpatient Care Unit	46	56,8
Kiara (COVID-care unit)	26	32,1
Ageusia symptom		
Ageusia	0	0
Non-ageusia	81	100

All the 81 participants (100%) who failed the fit test immediately passed the repeated fit test using the modified N95 sealed with plastic tape adhesive (MAR). After the participants used the modified adhesive respirator sealed with plastic tape adhesive for 4 h under normal working condition, 52 participants (64.2%) still passed the fit test (Table 2).

**Table 2:** Modified adhesive respirator (MAR) fit test results (n = 81).

Fit Test Results	Frequency	Percentage
Immediately after using MAR		
Fail	0	0,0
Pass	81	100,0
4 h using MAR		
Fail	29	35,8
Pass	52	64,2

No significant correlations were found between gender, age group, type of work and the use of modified adhesive respirators for 4 h (P = 0,056; P = 0.762; P = 0.196) (Table 3). However, interestingly bivariate analysis revealed a significant difference between workplace variations in Kiara (COVID-care Unit) and fit test results of participant after 4 h use of modified adhesive respirator (P = 0.001; RR = 3.81) (Table 3).

**Table 3:** Bivariate Analysis of Risk Factors and Fit Test Results of Participant using Modified Respirator with Plastic Tape Seal after 4 Hours.

Risk factors	Fit Test Result		*p*	RR	95% CI	
	Fail	Pass			Low	High
Gender			0,056	1,99	1,05	3,78
Male	8	5				
Female	21	47				
Age group			0,762	0,84	0,35	1,64
21–40 years	25	43				
41–54 years	4	9				
Type of job *)			0,196	2,35	0,77	7.17
Nurse	27	42				
Intern/Doctor	2	10				
workplace *)						
Kiara (COVID-care unit)	22	4				
Inpatient Care Unit	5	41	0,001**	3,81	1,79	8,09
ENT Specialist clinic***	2	7	0,321	0,49	0,11	2,19

Chi Square *) Fisher Exact **) *p* < 0,05. ***) Reference.

To further examine the data obtained from the results of bivariate analysis, the variables with P value < 0.25 (gender, type of work and workplace in Kiara) were analyzed using multivariate analysis using the Backward LR Logistics Regression test. The results of multivariate analysis using Logistics Regression Backward LR method found that the workplace in Kiara had the most significant influence on the fit test failing rate of participant using modified adhesive respirator for 4 h after being analyzed simultaneously with other risk factors (Table 4). Participants who work in Kiara (COVID-Care Unit) risk of failing fit test after using the modified adhesive respirator 14.70 times the incidence of participants in the outpatient clinic.

**Table 4:** Risk Factors Affecting Fit Test Failure of Participant using Modified Adhesive Respirator for 4 h.

Risk Variabile	Beta	P Value	OR	95% CI	
				Low	High
Kiara ward	2,69	0,000	14,70	4,77	45,33
Konstanta	−4,14

**Table 5:** Probability of Failing Fit Test using Modified Adhesive Respirator for 4 h.

Site	Probabilities
ENT Specialist clinic	1,57%
Inpatient Care Unit	19,00%
Kiara (Covid-care Unit)	77,56%

It can be seen from Table 5, that the probability of failing fit test after using the of the modified adhesive respirator for 4 h according to workplace (ENT Specialist clinic, Inpatient Care Unit, and Kiara (COVID-Care Unit) are 1.57%, 19,00%, and 77.56%, respectively.

## 4 Discussions

The risk of facial seal leakage in the use of Personal Protective Equipment (PPE), especially in the use of N-95 respirators, should be known and anticipated in the current COVID-19 pandemic era. The incompatibility of the shape of the respirator to the user's face will significantly affect the incidence of leakage of the respirator use [4,5]. The existence of a fit test procedure will be extremely helpful in detecting any leaks in the use of the respirator in question before being used in an infectious environment. In this study, we used the N-95 respirator type 1860 because this type of respirator was the only type of N-95 respirator with medical grade standards by NIOSH which was widely available in the Cipto Mangunkusumo General Hospital environment. This respirator has a layer to protect the wearer to prevent transmission of infection through droplets and splashes of blood or other liquid substances. In addition, it also serves to reduce aerosol exposure when the Aerosol Generating Procedure (AGP) is carried out. The aim of this study is to find a solution to meet the standard safety requirements for health workers, especially at the Cipto Mangunkusumo General Hospital environment to ensure the health care workers feel safer at work, particularly in the current pandemic era.

Every year many health workers failed the qualitative fit test, making them more vulnerable to the risk of face seal leakage. In N-95 respirators, most of the particles enter through the gap between the respirator and the face, this indicates that the importance of the density of the use of the respirator to the face is as important as increasing the effectiveness of the respirator's filtration [6].

The research participants were 92 health workers who agreed to perform a Bitrex Qualitative Fit test. It was found that from the total of 92 participants, 81 participants failed the test using the chosen N-95 respirator (3 M 1860 respirator). All the participants who failed the fit test was retested using a modified adhesive respirator (3 M, 1860 respirator sealed with Tegaderm® film dressing) and evaluated after 4 h of use under normal working condition. The sex distribution of the subjects in this study were mostly female as many as 68 subjects and male as many as 13 subjects. This was also found in a previous study by Wardhan et al. [2] who stated that in their study using a modified method of attaching N-95 respirators to the face using two-sided adhesive adhesives, 89% of the study population were women. This also supports the propose risk of facial seal leak in women due to the use of cosmetics and a lot of talking. The age grouping of health workers who became the research subjects was divided into 2 groups based on Reksodiputro M.H [7] who stated that changes in skin structure at the age of 40 years are mainly due to hormonal changes, thus underlie the division of age groups into age groups 20–40 years and 41–55 years.

The next variable is the type of work which was dominated by 69 nurses, 11 doctors and 1 student. This is in accordance with Al Thobaity A and Alshammari F33, who explained that the majority of health workers consisted of nurses who were sent to emergency hospitals during the COVID-19 pandemic. The workplace is dominated by subjects who worked in the Inpatient Building A as many as 46 subjects, Kiara 26 subjects and the Outpatient clinic as many as 9 subjects. This is done to maintain the safety of researchers and subjects, namely in the green zone where health workers used the N-95 type 1860 respirator in accordance with the recommendations issued by the Hospital Infection Prevention and Control Program at the CMGH.

All research subjects showed excellent results by showing a 100% success rate when evaluating the fit test was evaluated immediately after sealing. Subjects who had been declared not leaking, continued with the use of respirators that had been sealed for 4 h with daily work activities. This is based on the recommendation from WHO [8], which states that the use of an N-95 respirator for more than 4 h will cause discomfort to the user and should be avoided. The observation of the fit test after 4 h of use resulted in the majority of the subjects having no leakage (64.2%). This is similar to the outcome of a research by Wardhan et al. [2] which stated that the modified N-95 respirator with double-sided adhesive on the inner side of the respirator resulted in observations of 68% of subjects not leaking on the fit test shortly after gluing. Although respirators generally adhere strongly to the face, after using them for a while the attachment can be disrupted. Several factors, including physical activity, breathing rate, and body and head movements can affect the entry of particles through the gap between the respirator and the face [5]. Some studies also show that N-95 respirators fail to protect health workers when undergoing strenuous activities such as lifting heavy weights, running, and performing cardiopulmonary compression [9,10].

In Table 3, bivariate analysis of risk factors and N-95 respirator leakage after 4 h of sealing in Kiara was 3.81 times more compared to the leakage at outpatient clinic with a p value of 0.001. This is because when the researcher made the observations, the conditions of the room at Kiara were filled with COVID-19 patients with variations ranging from moderate to severe symptoms compared to working conditions at the outpatient clinic where the patient had milder symptom. In addition, researchers observed that the clothes worn can affect health workers at Kiara. These health workers wore hazmats made of synthetic materials with waterproof film-coated fabrics starting from levels 2 and 3, this can affect the health workers to sweat more than the health workers in ward Building A and outpatient clinic who wore hospital uniforms for nurses. and added cloth robes for doctors so that there is less sweat formation. Also, the temperature at Kiara's ward ranges from 17 to 25° Celsius with a humidity of 45–71%. The temperature in the Inpatient Room Building A ranged from 23 to 27° Celsius with a humidity of 35–57%. The temperature in the outpatient clinic ranged from 23 to 27° celsius with a humidity of 55–62%; thus it can affect a person's sweat production [11].

The use of plastic tape in this study has never been used in other studies, but in the preliminary study used another type of adhesive made from non-woven polyester (hypafix®). In the study, it was said that out of 100 subjects, there were 86 subjects who had leaks in the saccharin fit test with various types of N-95 respirators. Furthermore, sealing using hypafix around the N-95 respirator was carried out and a re-fit test was performed which resulted in a success rate of overcoming the leak up to 67%.

This study has added benefit for the health woven which can be seen from the success rate of sealing the N-95 type 1860 respirator in subjects who experienced a leak through a fit test which reached 100%. In addition, it can also be used as a solution to overcome leakage in 4 h of use in a workplace that was humid, such as the Outpatient clinic and Ward Building A. Especially for the use at Kiara, this type of adhesive could still be used with a note that the subject should pay more attention to the adhesiveness of this plastic tape seal so that it can still prevent leakage from the use of the N-95 respirator used.

The most important limitation in this study is none of the sealing technique for respirators had been standardized and validated before. No studies have tested the prototypes of modified mask with any adhesives. The modifications made may not be adequate to protect its user because these respirators and adhesives have not gone through a series of vigorous tests as a comercial respirator would. Several respirators have used new technologies to overcome face seal leak problem such as Novel FitsSeal adhesion technology by Wein Products (Los Angeles, CA) and FaceSeal Technologies (Toronto, Canada) which have products available in the market. Another limitation is that this study only uses a qualitative fit test Bitrex solutions; therefore, it can only detect the presence or absence of the subject's bitter taste ability and cannot distinguish if the subject has abnormalities in taste buds such as hypogeusia. Given the relatively small number of health care workers recruited due to scarcity of resources, caution must be exercised.

Respirator fit test is important for the safety of health care worker. Respirator only can provide respiratory protection to its user when used properly, which means no face-seal leakage is detected while using it under the fit test. The face-seal leakage largely contributes to the penetration of particles through respirator and could provide protection. Education about the importance of fit test and routine inspections needs to be carried out to identify and anticipate face-seal leaks, thus, could overcome the face-seal leak problem in the use of N-95 respirators. This would hopefully prevent transmission of COVID-19 to the health care workers. Frequent checks are required to ensure that the adhesives and respirators are still intact, especially in workplace with high workload and places where there is a higher occurrence of sweating. Further studies need to be conducted to determine the effectiveness of the use of plastic tape seals around the N-95 respirator to seal respirator to face when healthcare workers use it for more than 4 h in outpatient clinic or ward which was identified in this study as the place with less face-seal leakage incidents. Further works will need to be performed on what factors can affect respirator modification success in those working environment.

Further investigations need to be carried out to find suitable type of N-95 respirator and other forms of adhesive to overcome face seal leak problem in health care workers who works more than 4 h in an environment that was identified in this study as place with higher incident of face-seal leak. It is necessary for the hospital to encourage the respiratory protection program to provide a place to conduct fit test on a regular basis in high-risk places.

## 5 Conclusions

Although there is a need for sealing techniques for respirators had been standardized and validated before, the plastic tape had considerable effectiveness in sealing to overcome face seal leak on the N-95 type 1860 respirator.

## Annals of medicine and surgery

The following information is required for submission. Please note that failure to respond to these questions/statements will mean your submission will be returned. If you have nothing to declare in any of these categories then this should be stated.

## Please state any conflicts of interest

All authors must disclose any financial and personal relationships with other people or organisations that could inappropriately influence (bias) their work. Examples of potential conflicts of interest include employment, consultancies, stock ownership, honoraria, paid expert testimony, patent applications/registrations, and grants or other funding.

The author states no conflict of interest.

## Please state any sources of funding for your research

All sources of funding should be declared as an acknowledgement at the end of the text. Authors should declare the role of study sponsors, if any, in the collection, analysis and interpretation of data; in the writing of the manuscript; and in the decision to submit the manuscript for publication. If the study sponsors had no such involvement, the authors should so state.

This study didn't not receive any external funding.

## Ethical approval

Research studies involving patients require ethical approval. Please state whether approval has been given, name the relevant ethics committee and the state the reference number for their judgement.

The study had received ethical approval from Universitas Indonesia Faculty of Medicine's Health Medicine Research Ethics Committee (Approval Number: KET-171/UN2.F1/ETI/PPM.February 00, 2021).

## Consent

Studies on patients or volunteers require ethics committee approval and fully informed written consent which should be documented in the paper.

Authors must obtain written and signed consent to publish a case report from the patient (or, where applicable, the patient's guardian or next of kin) prior to submission. We ask Authors to confirm as part of the submission process that such consent has been obtained, and the manuscript must include a statement to this effect in a consent section at the end of the manuscript, as follows: “Written informed consent was obtained from the patient for publication of this case report and accompanying images. A copy of the written consent is available for review by the Editor-in-Chief of this journal on request”.

Patients have a right to privacy. Patients’ and volunteers' names, initials, or hospital numbers should not be used. Images of patients or volunteers should not be used unless the information is essential for scientific purposes and explicit permission has been given as part of the consent. If such consent is made subject to any conditions, the Editor in Chief must be made aware of all such conditions.

Even where consent has been given, identifying details should be omitted if they are not essential. If identifying characteristics are altered to protect anonymity, such as in genetic pedigrees, authors should provide assurance that alterations do not distort scientific meaning and editors should so note.

Yes, informed consent form and patient information sheet are made available in Indonesian for all the participants.

## Author contribution

Please specify the contribution of each author to the paper, e.g. study concept or design, data collection, data analysis or interpretation, writing the paper, others, who have contributed in other ways should be listed as contributors.

Mirta Hediyati Reksodiputro – Study concept or design, data analysis or interpretation, study supervision.

Harim Priyono – Study concept or design, data analysis or interpretation, study supervision.

Luthfi Ari Wibowo – Study concept or design, data analysis or interpretation, data collection, writing the paper.

Janny Bashiruddin – Study concept or design, data analysis or interpretation, study supervision.

Ratna Dwi Restuti – Data collection, writing the paper.

Dini W. Widodo – Data collection, writing the paper.

Dewi S. Soemarko - Study concept or design, data analysis or interpretation.

Joedo Prihartono – Data analysis or interpretation.

Prasandhya Astagiri Yusuf – Data analysis or interpretation.

Widayat Alviandi – Study concept or design, data analysis or interpretation.

Arie Cahyono – Data collection, writing the paper.

Semiramis Zislavsky – Data collection, wrtitting the paper.

Brastho Bramantyo – Study concept or design.

Fauziah Fardizza – Study concept or design.

## Registration of research studies

In accordance with the Declaration of Helsinki 2013, all research involving human participants has to be registered in a publicly accessible database. Please enter the name of the registry and the unique identifying number (UIN) of your study.

You can register any type of research at http://www.researchregistry.com to obtain your UIN if you have not already registered. This is mandatory for human studies only. Trials and certain observational research can also be registered elsewhere such as: ClinicalTrials.gov or ISRCTN or numerous other registries.

Name of Registry: Research Registry.

UIN: researchregistry7088.

## Guarantor

The Guarantor is the one or more people who accept full responsibility for the work and/or the conduct of the study, had access to the data, and controlled the decision to publish.

Mirta Hediyati Reksodiputro.
